# Tailored radiation dose according to margin width for patients with ductal carcinoma in situ after breast-conserving surgery

**DOI:** 10.1038/s41598-023-50840-8

**Published:** 2024-01-03

**Authors:** Hyunjung Kim, Tae Gyu Kim, Byungdo Park, Jeongho Kim, Si-Youl Jun, Jun Ho Lee, Hee Jun Choi, Chang Shin Jung, Hyoun Wook Lee, Jae Seok Lee, Hyun Yeol Nam, Seunghyen Shin, Sung Min Kim, Haeyoung Kim

**Affiliations:** 1 Departments of Radiation Oncology, Samsung Changwon Hospital, Sungkyunkwan University School of Medicine, Changwon, 630-522 South Korea; 2 Departments of Surgery, Samsung Changwon Hospital, Sungkyunkwan University School of Medicine, Changwon, South Korea; 3 Departments of Pathology, Samsung Changwon Hospital, Sungkyunkwan University School of Medicine, Changwon, South Korea; 4 Departments of Nuclear Medicine, Samsung Changwon Hospital, Sungkyunkwan University School of Medicine, Changwon, South Korea; 5 Departments of Internal Medicine, Samsung Changwon Hospital, Sungkyunkwan University School of Medicine, Changwon, South Korea; 6 Department of Radiation Oncology, Samsung Medical Center, Sungkyunkwan University School of Medicine, Seoul, South Korea

**Keywords:** Cancer, Medical research, Oncology

## Abstract

A 2 mm resection margin is considered adequate for ductal carcinoma in situ (DCIS). We assessed the effectiveness of a tailored radiation dose for margins < 2 mm and the appropriate margin width for high-risk DCIS. We retrospectively evaluated 137 patients who received adjuvant radiotherapy after breast-conserving surgery for DCIS between 2013 and 2019. The patients were divided into three– positive, close (< 2 mm), and negative (≥ 2 mm) margin groups. Radiation dose to the tumor bed in equivalent dose in 2 Gy fractions were a median of 66.25 Gy, 61.81 Gy, and 59.75 Gy for positive, close, and negative margin groups, respectively. During a median follow-up of 58 months, the crude rates of local recurrence were 15.0%, 6.7%, and 4.6% in the positive, close, and negative margin groups, respectively. The positive margin group had a significantly lower 5-year local recurrence-free survival (LRFS) rate compared to the close and negative margin groups in propensity-weighted log-rank analysis (84.82%, 93.27%, and 93.20%, respectively; *p* = 0.008). The difference in 5-year LRFS between patients with the high- and non-high-grade tumors decreased as the margin width increased (80.4% vs. 100.0% for margin ≥ 2 mm, *p* < 0.001; 92.3% vs. 100.0% for margin ≥ 6 mm, *p* = 0.123). With the radiation dose tailored for margin widths, positive margins were associated with poorer local control than negative margins, whereas close margins were not. Widely clear margins (≥ 2 mm) were related to favorable local control for high-grade DCIS.

## Introduction

The incidence of ductal carcinoma in situ (DCIS) of the breast has increased from 5.8 per 100,000 women in the 1970s to 32.5 per 100,000 presently, possibly because of increased diagnosis by screening^1^. Previous randomized trials have reported that adjuvant radiotherapy halves local recurrence after breast-conserving surgery^2^. Therefore, the treatment of DCIS generally consists of breast-conserving surgery followed in most cases by adjuvant radiotherapy. Studies of mastectomy specimens have demonstrated that although most cases of DCIS are unifocal, 8% of patients with DCIS have a multifocal growth pattern with gaps of more than 10 mm^3,4^. In patients who underwent breast-conserving surgery alone without adjuvant radiotherapy, a surgical margin of < 10 mm was associated with higher local recurrence^5^. Considering the growth pattern of DCIS and high local recurrence with close surgical margins, appropriate margin width for breast-conserving surgery is important.

A meta-analysis of appropriate margin widths in patients who underwent both breast-conserving surgery and adjuvant radiotherapy demonstrated that local recurrence was significantly reduced at 2 mm compared with 0 (no ink on tumor) or 1 mm thresholds. However, there were no significant differences in the odds of local recurrence between patients with margins of 2 mm, 3 mm, 5 mm, and 10 mm (odds ratio compared to margin < 2 mm: 0.51, 0.42, and 0.60, respectively)^6,7^. Therefore, the Society of Surgical Oncology/American Society of Radiation Oncology (SSO/ASTRO) guidelines recommend using a 2 mm margin as the standard for adequate resection of DCIS treated with adjuvant radiotherapy^8^. The routine practice of obtaining negative margin widths > 2 mm is not recommended. The guidelines also state that the increased risk of local recurrence in patients with positive margins, defined as ink on DCIS, is not nullified by adjuvant radiotherapy.

However, in the studies that served as the basis for these guidelines, only some patients received boost radiotherapy^2,9,10^. Moreover, the boost dose was the same in patients with close/positive margins and those with negative margins. Higher radiation doses may nullify the negative effects of close/positive margins on local control in patients with DCIS^11^. However, research on this topic is insufficient^12^. The guidelines also do not indicate whether a 2 mm margin is appropriate for patients with unfavorable factors. Our institution uses a higher radiation dose for close/positive margins, with a dose-escalation policy based on the margin width.

This study aimed to assess the effectiveness of an increased radiation dose for close/positive margins by comparing local recurrence among patients with negative, close, and positive margins. We also investigated whether the 2 mm threshold was appropriate for patients with a high risk of local recurrence.

## Methods

After receiving Institutional Review Board approval, we identified 192 patients who received adjuvant radiotherapy after breast-conserving surgery for DCIS between April 2013 and December 2019 at Samsung Changwon Hospital, Changwon, South Korea. The patients’ medical records were retrospectively reviewed. The inclusion criteria were breast-conserving surgery, ≥ 6 months of follow-up, and completion of scheduled radiotherapy. Patients with synchronous invasive or micro-invasive cancers were excluded. Patients with histories of other malignancies or recurrent DCIS were also excluded. A total of 137 patients who met the selection criteria were analyzed.

Pre-treatment imaging included ultrasonography, mammography, or magnetic resonance imaging. All patients underwent breast-conserving surgery followed by adjuvant radiotherapy for DCIS with curative intent. Endocrine therapy was administered to patients with hormone receptor-positive disease. Post-excision mammograms were obtained for patients presenting with mammographic calcifications. Radiotherapy was planned using standard breast board positioning and computed tomography simulation. Radiotherapy was delivered with two tangential fields using 6 MV photons. All patients received whole-breast radiotherapy at 50 Gy in 25 fractions or 50.4 Gy in 28 fractions schedules. Sequential boost radiotherapy using an electron beam was delivered after whole-breast radiotherapy, except for eight patients with wide negative margins and low risk, following the clinician’s decision. The boost field was expanded by a 2 cm margin around the surgical clips and/or the tumor bed, as shown by simulation computed tomography. The boost dose schedule followed our dose-escalation policy according to margin width–15 Gy in five fractions for positive margins; 10.5 Gy in three fractions for margin width < 3 mm; no boost or 9 Gy in three fractions for margin width ≥ 3 mm. Positive margins were defined as “ink on tumor.” To analyze the adequacy of the 2 mm margin, clear margins were grouped into ≥ 2 mm (negative) or < 2 mm (close) margins according to SSO/ASTRO guidelines^13^. Accordingly, the boost doses were 15 Gy/five fractions, 10.5 Gy/three fractions, and 0–10.5 Gy/three fractions in the groups with positive margins, < 2 mm, and ≥ 2 mm negative margins, respectively (total equivalent dose in 2 Gy fractions (EQD2) to the tumor bed (α/β = 10 Gy): median 66.25 Gy (range: 65.81–66.25 Gy), 61.81 Gy (range: 61.37–61.81 Gy), and 59.75 Gy (range: 49.56–61.81 Gy), respectively). All patients were followed up regularly with clinical examination and bilateral mammography every 6 months for the first 5 years and annually thereafter. Local recurrence was defined as any pathologically confirmed recurrence (invasive or DCIS) of the ipsilateral breast. The time to event was calculated from the date of surgery.

A statistician performed all statistical analyses. The characteristics of patients with positive, close, and negative margins were compared using Pearson’s chi-square test. To reduce the effects of selection bias between groups, significant differences in characteristics were adjusted using the inverse probability of treatment weights (IPTW) and propensity scoring. Local recurrence-free survival (LRFS) was plotted using the Kaplan–Meier method and compared using the log-rank test. The prognostic factors for local recurrence were evaluated using the Cox proportional hazards model. Hazard ratios (HRs) and 95% confidence intervals (CIs) were determined. Factors with *p* < 0.1 in the univariate analyses were included in the multivariable analysis. Binary logistic regression analysis was used to evaluate the association between margin width and local recurrence. All statistical analyses were performed using STATA (version 15.1; Stata Corp., College Station, TX, USA). Statistical significance was defined as a two-sided *p*-value ≤ 0.05.

### Ethical approval and consent to participate

The Institutional Review Board of Samsung Changwon Hospital approved this retrospective study (SCMC 2022-11-001) and waived the requirement for patient informed consent. All procedures involving human participants followed the ethical standards of the institutional and/or national research committee and the 1964 Declaration of Helsinki and its later amendments or comparable ethical standards.

## Results

The median follow-up period for the 137 patients was 58 months (range 6–117). The median follow-up period was 59.5 months (range 8–113) for 20 patients with positive margins, 57.5 months (range 20–114) for 30 patients with close margins, and 57 months (range 6–117) for 87 patients with negative margins. The median time to local recurrence was 28 months (range 9–47). Local recurrence occurred in three, two, and four patients in the positive, close, and negative groups, respectively (crude rates of 15.0%, 6.7%, and 4.6%, respectively). Of the nine local recurrences, two were invasive ductal carcinomas and seven were DCIS.

### Comparison of local recurrence between positive, close, and negative margin groups

**Figure 1:**
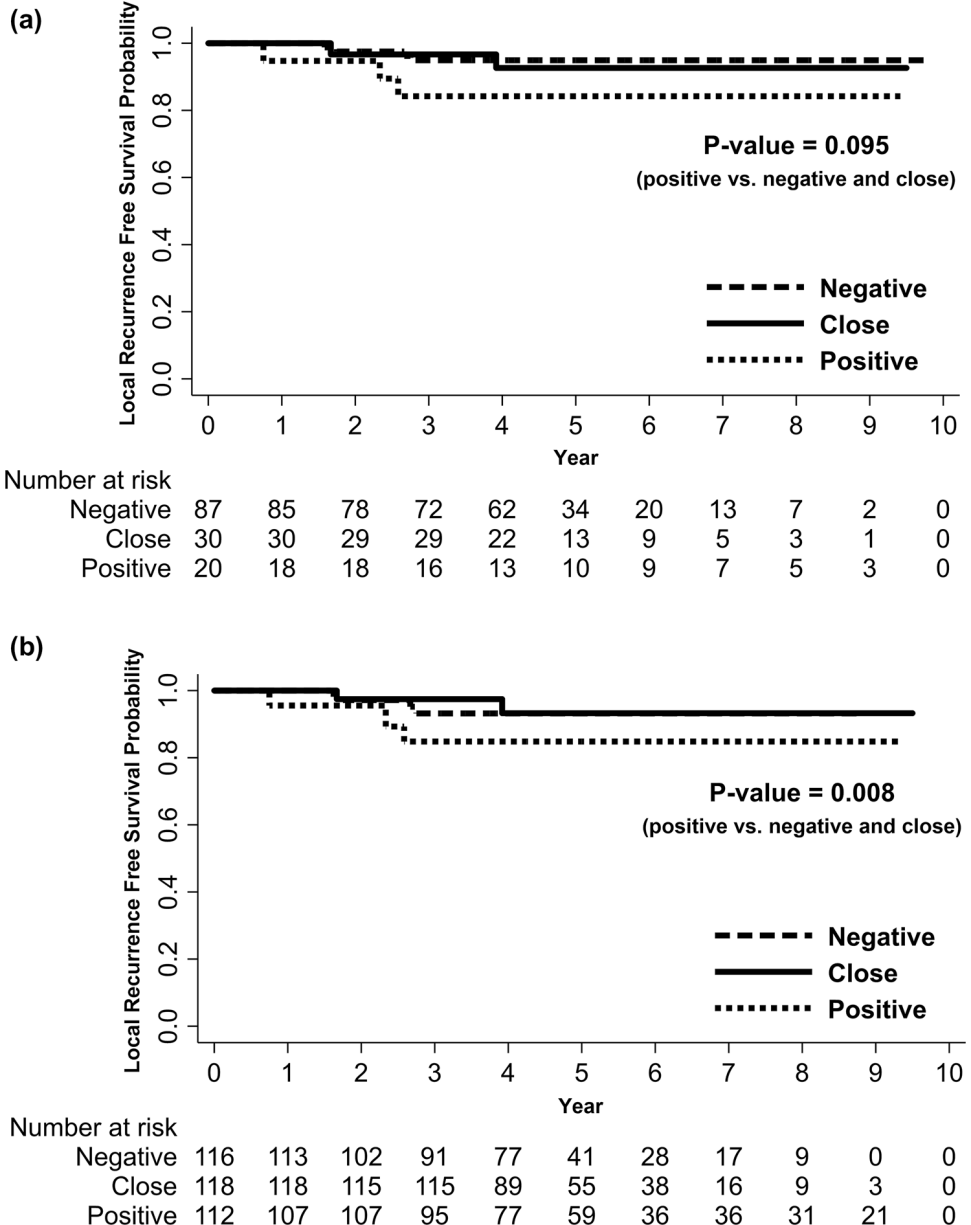
Local recurrence-free survival according to margin status before (**a**) and after (**b**) a propensity-weighted log-rank analysis.

Patient characteristics are shown in Table 1. There were no significant differences in patient characteristics, including age, symptomatic presentation, necrosis, multifocality, nuclear grade, Ki-67, hormone receptor status, and human epidermal growth factor receptor 2 (HER2) status, among the positive, close, and negative margin groups. However, the positive or close margin group had significantly more tumors > 10 mm in size than the negative margin group (*p* = 0.034). 5-year LRFS rate was 92.87% in all patients. There was no significant difference in LRFS among the three groups (*p* = 0.239) or between the negative and close margin groups (*p* > 0.999) but the positive margin group had inferior LRFS compared to the close and negative margin groups, however, this was statistically insignificant (5-year LRFS rates: 84.21%, 92.64%, and 94.93%, respectively; *p* = 0.095; Fig. 1a).

**Table 1:** Patient characteristics according to margin width. *IPTW* inverse probability of treatment weight, *HER2* human epidermal growth factor receptor 2.

Characteristics	Overall (%) (n = 137)	Positive (%) (n = 20)	Close (%) (n = 30)	Negative (%) (n = 87)	*p*-value	
					Before IPTW	After IPTW
Age (years)						
≤ 50	82 (59.85)	14 (7000)	15 (50.00)	53 (60.92)	0.404	0.428
> 50	55 (40.15)	6 (30.00)	15 (50.00)	34 (39.08)		
Symptomatic presentation						
Yes	35 (25.55)	5 (25.00)	4 (13.33)	26 (29.89)	0.200	0.910
No	102 (74.45)	15 (75.00)	26 (86.67)	61 (70.11)		
Tumor size						
≤ 10 mm	58 (42.34)	5 (25.00)	9 (30.00)	44 (50.57)	0.034	0.907
> 10 mm	79 (57.66)	15 (75.00)	21 (70.00)	43 (49.43)		
Necrosis						
Yes	83 (60.58)	10 (50.00)	19 (63.33)	54 (62.07)	0.573	0.096
No	54 (39.42)	10 (50.00)	11 (36.67)	33 (37.93)		
Multifocality						
Yes	36 (26.28)	7 (35.00)	10 (33.33)	19 (21.84)	0.295	0.414
No	101 (73.72)	13 (65.00)	20 (66.67)	68 (78.16)		
Nuclear grade						
Low-Intermediate	103 (75.18)	15 (75.00)	23 (76.67)	65 (74.71)	0.977	0.097
High	34 (24.82)	5 (25.00)	7 (23.33)	22 (25.29)		
Ki-67 (%)						
≤ 10	84 (61.31)	11 (55.50)	18 (60.00)	55 (63.22)	0.523	0.453
> 10	35 (25.55)	4 (20.00)	9 (30.00)	22 (25.29)		
Unknown	18 (13.14)	5 (25.00)	3 (10.00)	10 (11.49)		
Hormone receptor						
Positive	103 (75.18)	14 (70.00)	22 (73.33)	67 (77.01)	0.779	0.163
Negative	34 (24.82)	6 (30.00)	8 (26.67)	20 (22.99)		
HER2						
Positive	40 (29.20)	4 (20.00)	12 (40.00)	24 (27.59)	0.605	0.059
Negative	89 (64.96)	15 (75.50)	17 (56.67)	57 (65.52)		
Unkown	8 (5.84)	1 (5.00)	1 (3.33)	6 (6.90)

After compensating for the main effect of tumor size on selection bias using the IPTW method, LRFS was compared among the positive-, close-, and negative-margin groups using propensity-weighted log-rank analysis (Table 1. and Supplementary Table 1). The positive margin group had significantly worse LRFS rates than the close and negative margin groups (5-year LRFS rates of 84.82%, 93.27%, and 93.20%, respectively; *p* = 0.008; Fig. 1b). There was no significant difference in the LRFS rate between the negative and close margin groups (*p* > 0.999).

### Appropriate margin width for high-risk subgroup

Univariable analyses revealed that a high nuclear grade, negative hormone receptor status, and positive HER2 status were significant risk factors for local recurrence (*p* = 0.003, 0.005, and 0.030, respectively; Table 2). A tumor size > 10 mm was associated with a higher rate of local recurrence but this trend was not statistically significant (*p* = 0.089). In a multivariable analysis including these factors, the nuclear grade was the only independent significant factor for local recurrence (*p* = 0.049).

**Table 2:** Risk factors for local recurrence. *HR* hazard ratio, *CI* confidence interval, *HER2* human epidermal growth factor receptor 2.

Characteristics	Univariable		Multivariable	
	HR (95% CI)	*p*-value	HR (95% CI)	*p*-value
Age (years)				
≤ 50	Reference			
> 50	0.75 (0.19, 3.00)	0.685		
Symptomatic presentation				
Yes	0.86 (0.18, 4.13)	0.848		
No	Reference			
Tumor size				
≤ 10 mm	Reference		Reference	
> 10 mm	6.08 (0.76, 48.65)	0.089	4.04 (0.50, 32.70)	0.191
Necrosis				
Yes	5.21 (0.65, 41.66)	0.120		
No	Reference			
Multifocality				
Yes	0.77 (0.16, 3.72)	0.749		
No	Reference			
Nuclear grade				
Low-Intermediate	Reference		Reference	
High	11.19 (2.33, 53.87)	0.003	5.86 (1.01, 34.11)	0.049
Ki-67 (%)				
≤ 10	Reference			
> 10	2.80 (0.75, 10.41)	0.125		
Hormone receptor				
Positive	0.14 (0.03, 0.55)	0.005	0.48 (0.09, 2.72)	0.408
Negative	Reference		Reference	
HER2				
Positive	4.65 (1.16, 18.59)	0.030	1.15 (0.27, 6.81)	0.717
Negative	Reference		Reference	
Resection Margin				
< 2 mm	Reference			
≥ 2 mm	0.48 (0.13, 1.79)	0.273		

**Figure 2:**
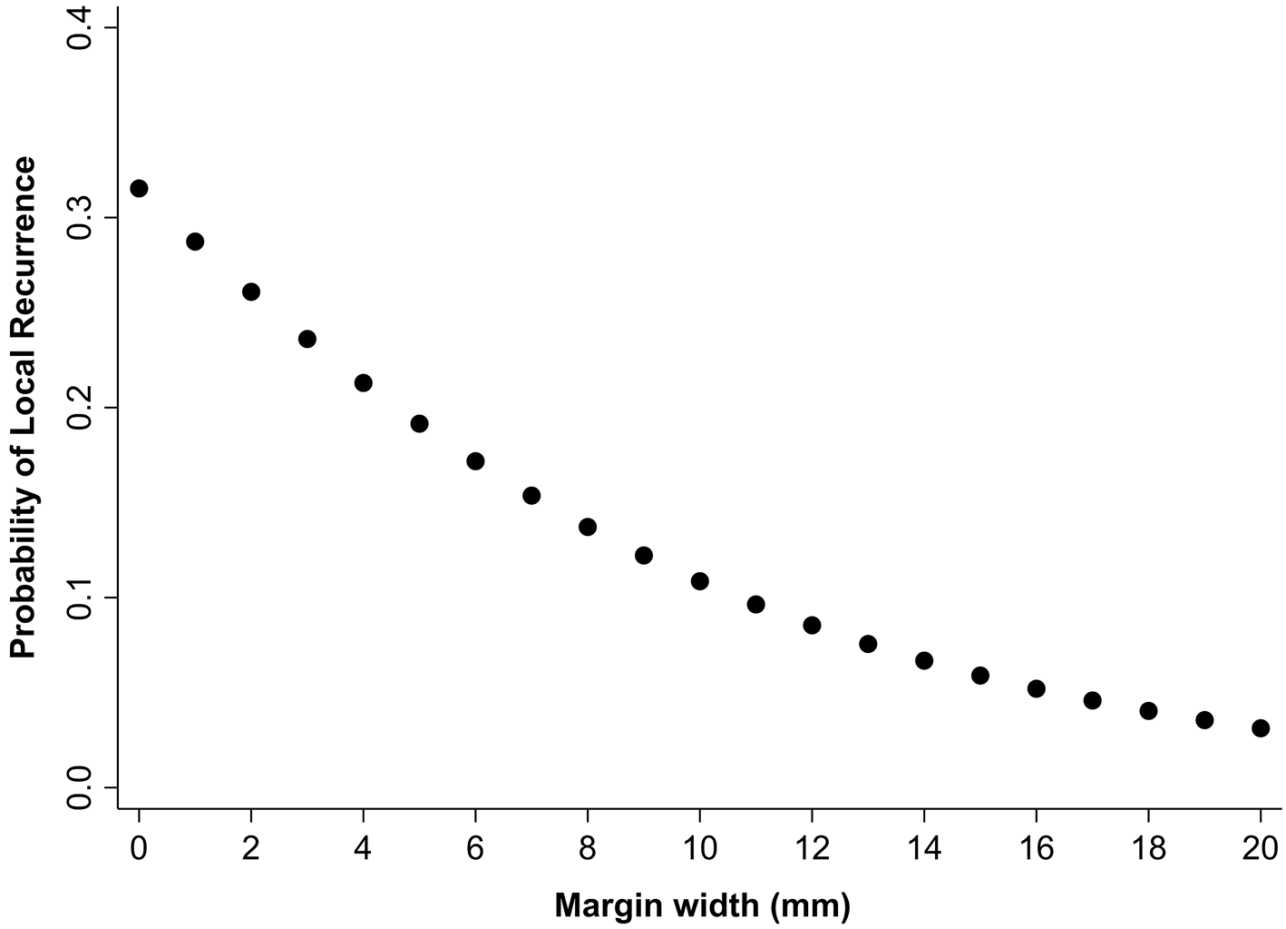
Estimated probability of local recurrence according to margin width in patients with high-grade tumor.

To evaluate the appropriate margin width for high-grade tumors, local recurrence-free survival was compared between the high- and non-high-grade groups according to the margin width (Table 3). In patients with a margin width ≥ 2 mm, the high-grade group had a significantly inferior LRFS than the non-high-grade group (*p* < 0.001). The difference in survival rates between the high- and non-high-grade groups decreased as the margin width increased. The difference in survival between the two grade groups disappeared in patients with a margin width ≥ 6 mm (*p* = 0.123). Binary logistic regression analysis in the high-grade group showed that the probabilities of local recurrence were 19% and 11% for margin widths of 5 mm and 10 mm, respectively. The probability of local recurrence decreased as the margin width increased (Fig. 2).

**Table 3:** Comparison of local recurrence-free survival rates between high grade and non-high-grade tumor groups according to margin width.

Margin width (mm)	5-year local recurrence-free survival rate (%)		*p*-value
	High grade	Non-high grade	
< 2	75.00	94.59	0.056
≥ 2	80.42	100.00	< 0.001
≥ 3	82.64	100.00	0.003
≥ 4	80.36	100.00	0.003
≥ 5	86.15	100.00	0.024
≥ 6	92.31	100.00	0.123
≥ 7	91.67	100.00	0.120
≥ 8	91.67	100.00	0.134
≥ 9	90.91	100.00	0.124
≥ 10	90.91	100.00	0.132

## Discussion

In this study, patients with negative (≥ 2 mm) and close (< 2 mm) margins showed similar local recurrence rates with boost dose-escalation according to margin width. However, radiation doses > 65 Gy EQD2 did not nullify the negative effect of positive margins on local control. In patients with high-grade DCIS, more widely clear margins were associated with lower recurrence rates compared to 2 mm margins, which were suggested as an appropriate width for DCIS. Margin width ≥ 6 mm could reduce the difference in local control between patients with high-grade and non-high-grade tumors to a non-significant level.

Several studies have investigated whether radiotherapy can replace re-excision for the treatment of positive margins, defined as ink on DCIS. In a meta-analysis of randomized trials by the Early Breast Cancer Trialists’ Collaborative Group (EBCTCG), patients with positive margins had a two-fold higher risk of local recurrence than those with negative margins despite receiving whole-breast radiotherapy (10-year local recurrence rate of 24.2% vs. 12.0%)^2^. Most of the trials included in this study involved whole-breast radiotherapy (50 Gy in 25 fractions), and only some patients (0–9%) received boost radiotherapy. In a meta-analysis of 20 studies by Marinovich et al., negative margins had an odds ratio of 0.45–0.37 for local recurrence compared to those with positive margins, even when evaluating only study cohorts with adjuvant radiotherapy^7^. Accordingly, the SSO/ASTRO guidelines state that a positive margin is associated with a significant increase in local recurrence and that the increased risk is not nullified by radiotherapy^8^. However, some retrospective studies have demonstrated favorable outcomes with high-dose radiotherapy for positive DCIS margins, suggesting the substitutability of radiotherapy instead of re-excision. Lee et al. analyzed 368 patients with positive margins on DCIS after breast-conserving surgery^14^. They compared 24 patients who underwent re-excision and radiotherapy to 344 patients who received high-dose radiotherapy only (total dose: 65 Gy). The local recurrence rates were 4.2 and 1.2% in the two groups, respectively, with no significant differences (*p* = 0.262). Monteau et al. analyzed 208 patients with close (< 2 mm) or positive margins after breast-conserving surgery for DCIS, of whom 61 underwent re-excision before radiotherapy and 147 received high-dose radiotherapy (median total dose: median 67 Gy)^12^. The 7-year locoregional recurrence rates were 9.6% and 9.3% in patients who did and did not undergo re-excision, respectively (*p* = 0.9331). A study of 125 patients with positive margins (in situ disease only in 85%) after breast-conserving surgery for breast cancer conducted by Suzuki et al. showed favorable local control in patients who received a sufficient boost dose (total dose: 66 Gy; 10-year local recurrence-free survival rate:95%)^15^. These retrospective studies suggest that the substitutability of radiotherapy for the re-excision of positive margins is characterized by the use of a high-dose boost. In this study, a high-dose boost similar to that used in previous studies was applied to the positive margin (median dose: 66.25 Gy EQD2). Even after a high-dose boost, patients with positive margins showed worse local control than those with negative or close margins. The results of this study support the SSO/ASTRO guidelines that the increased risk in patients with positive margins is not nullified by radiotherapy.

Another important issue is whether radiotherapy contributes to sufficient local control in patients with negative but close margins (< 2 mm). A meta-analysis by Marinovich et al. showed significant reduction in odds ratio for 2 mm (0.51) relative to > 0 or 1 mm, in which 70.9% of the cohort received boost radiotherapy (median total dose: 60 Gy)^7^. A statewide population-based cohort study of 559 patients showed that close margins (< 2 mm) were associated with an increased risk of recurrence compared to margins ≥ 2 mm whether patients received radiotherapy (HR, 1.98; CI, 0.87–4.54) or not (HR, 1.32; CI, 0.27–6.49)^16^. The radiation doses used in this study have not been reported. However, a retrospective study of 2996 patients by Van Zee et al. showed no significant difference in the risk of recurrence with any margin threshold among patients receiving radiotherapy (*p* = 0.67, 0.96, and 0.70 for positive vs. tumor not on ink, ≤ 2 mm vs. > 2 mm, and ≤ 10 mm versus > 10 mm, respectively)^17^. The radiation dose used in this study has not been reported, too. In a study of 149 patients who underwent breast-conserving surgery for DCIS, there was no significant difference in locoregional recurrence between patients with < 2 mm and ≥ 2 mm negative margins who underwent radiotherapy (10-year locoregional recurrence rate: 4.8% versus 3.3%, respectively; *p* = 0.72)^18^. Radiotherapy to the tumor bed consisted of 64 Gy in 32 fractions for patients with close (< 2 mm) margins and 60 Gy in 30 fractions for patients with wider negative margins. In our study, there was no significant difference in local recurrence between patients with margins < 2 mm and those with margins ≥ 2 mm after a tailored boost according to the margin width. Taken together, re-excision when managing negative, but close margins remains controversial. However, several studies have demonstrated the possibility of a tailored radiation boost to attenuate the notorious effects of close margins on local recurrence.

High-grade DCIS has a higher risk of recurrence, progression to invasive cancer, and breast cancer-related death than low-grade DCIS^19–21^. Growth rates have increased with increasing grades of DCIS, as evidenced by the analysis of mammographic findings (1.8, 4.2, and 7.1 mm per year for low, intermediate, and high grades, respectively)^22^. In this study, there was no significant difference in the local recurrence rates between patients with close margins and those with negative margins in the entire cohort. However, in the high-grade DCIS group, the recurrence rate decreased when the margin width increased up to 6 mm. To the best of our knowledge, this is the first study to report that wide clear margins of > 2 mm are associated with lower recurrence rates in patients with high-grade DCIS. It could be inferred that patients with high-grade DCIS may have more remnant DCIS foci around the tumor bed than those with low-grade DCIS. High-grade DCIS may require more widely clear resection margins or an enhanced radiation boost, such as a larger field or dose escalation. To verify the findings of this study in terms of the margins for high-grade DCIS, a larger, well-designed study is required.

This study has several limitations owing to its retrospective nature. First, detailed information, such as the direction and extent (focal or extensive) of positive margins that could influence local recurrence, was not available. Second, toxicity according to dose escalation was not evaluated because of insufficient medical records. Third, the follow-up period of this study was relatively short compared with that of other studies on DCIS. Given the considerable long-term recurrence risk of DCIS, a longer follow-up period may alter the outcomes.

In conclusion, a negative but close (< 2 mm) margin did not worsen the local control with a tailored radiation dose in this study. However, a positive margin worsened local control, even with higher radiation doses > 65 Gy EQD2. In patients with high-grade DCIS, widely clear margins of > 2 mm were associated with lower recurrence rates in the setting of a boost. However, further prospective studies are required to verify these findings.

### Supplementary Information

Supplementary Table 1.

## Data Availability

The data supporting the findings of this study are available from the corresponding author upon reasonable request.
