# Supplementary material for: Tailored radiation dose according to margin width for patients with ductal carcinoma in situ after breast-conserving surgery
Source: Sci Rep. 2024 Jan 3;14:300. doi: 10.1038/s41598-023-50840-8 (PMC10761984; doi:10.1038/s41598-023-50840-8)
Supplement: Supplementary file 1 — Supplementary Table 1. [file 41598_2023_50840_MOESM1_ESM.docx]

| Negative vs. Close | IPTW variance ratio | |
| --- | --- | --- |
|  | Raw | Weighted |
| Age (≤50 vs. >50) | 1.076 | 0.969 |
| Symptomatic Presentation (Yes vs No) | 0.510 | 0.994 |
| Size (≤10 vs. >10) | 0.710 | 0.926 |
| Necrosis (Yes vs No) | 0.924 | 1.075 |
| Multifocality (Yes vs No) | 1.452 | 0.934 |
| Ki-67(≤10 vs. >10) | 1.116 | 0.875 |
| negative vs. positive | | |
| Age (≤50 vs. >50) | 0.988 | 0.901 |
| Symptomatic Presentation (Yes vs No) | 1.185 | 0.916 |
| Size (≤10 vs. >10) | 0.490 | 0.966 |
| Necrosis (Yes vs No) | 1.097 | 1.020 |
| Multifocality (Yes vs No) | 1.618 | 1.107 |
| Ki-67 (≤10 vs. >10) | 1.013 | 0.871 |

Supplementary Table1. Variance ratio of covariate balance. *IPTW* inverse probability of treatment weight.
